# How Useful Are Early Economic Models? Comment on "Problems and Promises of Health Technologies: The Role of Early Health Economic Modelling"

**DOI:** 10.15171/ijhpm.2019.119

**Published:** 2019-11-23

**Authors:** James Love-Koh

**Affiliations:** Centre for Health Economics, University of York, York, UK.

**Keywords:** Early Model, Cost-Effectiveness Analysis, Economic Evaluation, Decision Modelling

## Abstract

Early economic modelling has long been recommended to aid research and development (R&D) decisions in medical innovation, although they are less frequently published and critically appraised. A review of 30 innovations by Grutters et al provides an opportunity to evaluate how early models are used in practice. The evidence of early models can be used to inform two types of decision: to continue development ("stop or go") or to alter future R&D activities. I argue that early models have limited use in stop or go decisions, as less resource and data undermine the reliability of the models’ indicative estimates of cost-effectiveness. Whilst they are far more useful for informing future R&D directions, the best techniques available from statistical decision science, such as value of information analysis, are not regularly used. It is highly recommended that early models adopt these methods to best deal with uncertainty, quantify the potential value of further research, identify areas of study with the greatest potential benefit and generate recommendations on study design and sample size.

## Introduction

The development of innovative healthcare products such as pharmaceuticals and medical devices is highly resource intensive, involving billions of dollars.^1^ Public purchasers are the most common procurers of these products, with more than half of global health expenditure publicly funded.^2^ Manufacturers therefore have an obvious interest in trying to identify the social value of potential investments as early as possible in order to maximize revenue. Early health economic modelling is often proposed to aid investment decisions by estimating the expected costs and health benefits generated by an innovation, compared with current standards of care.

Early modelling has been recommended for at least 20 years.^3,4^ However, compared with more typical, later-stage decision models that are developed to inform adoption and reimbursement decisions (hereafter ‘late-stage models’), the details of early models are less frequently published. The recent retrospective analysis of 32 early models by Grutters and colleagues is therefore a welcome addition to this relatively under researched area.^5^ Their sample covers a variety of product development stages and clinical areas, with the vast majority commissioned by medical device companies. There is explicit focus on the early economic models themselves, rather than a broader early health technology assessment (HTA) process in which additional factors such as regulatory environment and safety profile are deliberated.^6^ ‘Early’ is defined as being any point before healthcare payers are making decisions about whether or not to adopt the intervention – the traditional point at which decision models are developed.

Grutters set out to address two questions relating to early models: (*i* ) how useful the models were in establishing potential cost-effectiveness and (*ii* ) exploring how the results affected the subsequent design or implementation of the intervention. Another way of framing these questions is whether the early model informed decisions about whether to (*i* ) continue development (“stop or go”) or (*ii* ) alter future research and development (R&D) activities (ie, by focusing additional research on promising patient groups). Since the principal motivation for decision modelling is to inform decisions, I agree that these are the principal justifications for developing early models. I argue that early models are far better at answering (*ii* ) than (*i* ).

## Stop or Go?

“Potential” cost-effectiveness is a low bar that a vast majority of early models clear. Every intervention in Grutters’ sample demonstrated some scenario in which it was cost-effective, as did an earlier smaller review by Markiewicz and colleagues.^7^ This is not surprising. First, compared with late-stage models, early models are programmed with less complete data of lower quality and higher uncertainty. Being built at an earlier phase of product development means that fewer trials will have been conducted, fewer patients treated and fewer outcomes monitored. Exploratory analyses of cost-effectiveness that accommodate this lack of data such as threshold or headroom analysis are therefore required.^8^ Testing large variations in critical drivers of cost-effectiveness, particularly the treatment’s cost and effectiveness, mean that the likelihood of some combination of parameters producing a cost-effective outcome is inherently high.

Developers of healthcare innovations may even have valid reasons for continuing development of products even if early models suggest that they will not be cost-effective. Every model is designed to address a particular decision problem; aspects include the perspective of the analysis (ie, health sector, multi-sector or societal), comparator interventions, the patient population and the relevant outcome measures. A stop recommendation from an early model could reflect different decision problems using scenario analysis, but for practical and technical reasons it may not be able to reproduce scenarios for all potential healthcare markets that an innovation has access to.

Last, early models are generally less well-resourced than late-stage models. The incentives for investing more in the latter are much stronger as they are a formal requirement in many countries’ HTA frameworks and can therefore directly influence the profitability of an intervention.^9^ These resources help the model maintain methodological standards set out by an agency’s reference case^10^ when it is subject independent critical appraisal in the HTA process. The relative under-resourcing of early models may lead to suboptimal modelling approaches or model structures being adopted, producing less reliable estimates of cost-effectiveness. All of Grutters’ early models, for instance, use more simplistic cohort Markov and decision tree structures. Whilst such approaches can often be appropriate,^11^ more advanced simulation methods may be overlooked by early modellers for budgetary reasons even when they are the preferred option.

## Develop Differently

The case for using early models to inform the direction of R&D of an intervention is much stronger. Grutters et al highlight several different ways in which their models were used for this purpose, such as highlighting the characteristics of a diagnostic test that most influenced cost-effectiveness or identifying an intervention’s optimal position in the disease treatment pathway.

Decision modelling techniques can also highlight fruitful directions for further research – research that investigates promising patient subgroups or reduces uncertainty around the value of the intervention. Grutters et al limit their exploration of uncertainty to deterministic sensitivity analysis; analysing the effects of varying the values of one or two parameters at a time. Although useful, they fall short of the methodological standard of probabilistic sensitivity analysis and value of information analysis that are uniformly recommended in the literature for early models.^4,12-14^ These analyses investigate the combined parameter uncertainty within the model, quantify the potential value of further research, identify areas of study with the greatest potential benefit and generate recommendations on study design and sample size.^15,16^

The lack of probabilistic analysis is acknowledged by Grutters et al and justified on the basis that it “is difficult to quantify the (non-statistical) uncertainty… in [terms of probability] distributions,” favouring the likes of tornado plots when assessing the impacts of the model parameters on results. However, deterministic sensitivity analysis and tornado plots still require a range of values that each parameter is to be varied over. If this range is not specified according to uncertainty (ie, through a probability distribution), then the influence of that parameter on cost-effectiveness will also be a function of a potentially *ad hoc* range selection. A solution to this issue is to estimate parameter values and/or probability distributions using structured expert elicitation methods, a number of which can be used depending upon the type of intervention or parameter under consideration.^17^ The use of structured expert elicitation would not only improve the validity of deterministic sensitivity analysis; it will also facilitate analytically preferable probabilistic methods and value of information analysis. Using these techniques will require more resource for early models, however, which may not be forthcoming due to the incentive problems noted above.

## Concluding Remarks

Grutters et al are right to conclude that their early models provide insight into the potential cost-effectiveness and associated uncertainty of their sample of innovations. However, this support comes with several caveats. The insights into potential cost-effectiveness are rather limited due to the exploratory nature of the analysis and may really only be truly useful when development decisions involve selecting a limited number of innovations from a portfolio, such as in a real options model.^18^ The insights into the drivers of cost-effectiveness and uncertainty, although much more worthwhile for R&D decision-making, could be greatly enhanced by using the best techniques available from statistical decision science. Similar conclusions have been reached by Abel et al during a review of early models of diagnostics.^19^

The focus of Grutters and of this article has been on how the outputs of early models *can* be used in R&D decisions. A related empirical question that should be the subject of future inquiry is whether those outputs *should* be used. One way of approaching this question is to compare the results of an early model with those of a late-stage model produced with greater resource and populated with better data. Such comparisons would be possible using an iterative Bayesian modelling process advocated by many in the literature,^3,13,14^ in which a model is regularly updated throughout the product development cycle. However, there is little evidence suggesting that this approach is being implemented, leaving this validation question around early models more challenging to address. Given the scarcity of published research on early models, Grutters et al deserve credit for at least shedding some light on this frequently private and internal process.

## Acknowledgments

Thanks to Sean Gavan, Matthew Taylor, and Richard Cookson for their many helpful comments and suggestions while drafting this article.

## Ethical issues

Not applicable.

## Competing interests

Author declares that he has no competing interests.

## Author’s contribution

JLK is the single author of the paper.
